# *“If it’s a broad spectrum, it can shoot better”:* inappropriate antibiotic prescribing in Cambodia

**DOI:** 10.1186/s13756-016-0159-7

**Published:** 2016-12-20

**Authors:** Chhorvoin Om, Frances Daily, Erika Vlieghe, James C. McLaughlin, Mary-Louise McLaws

**Affiliations:** 1 School of Public Health and Community Medicine, UNSW Medicine, UNSW, Level 3 Samuels Building, Sydney, NSW 2052 Australia; 2 Diagnostic Microbiology Development Program, # 23 (First Floor), Street 310, BKK 1, Khan Chamcar Morn, Phnom Penh, Cambodia; 3 Institute of Tropical Medicine, Nationalestraat 155, Antwerp, 2000 Belgium

**Keywords:** Antibiotic resistance, Infection control, Preventive, Microbiology, Qualitative study, Prescribing habit

## Abstract

**Background:**

Cambodia is affected by antibiotic resistance but interventions to reduce the level of resistance require knowledge of the phenomena that lead to inappropriate prescribing. We interviewed physicians working in public hospitals to explore the drivers of inappropriate antibiotic prescribing.

**Methods:**

Hospitals participating in a knowledge, attitudes and practices survey prior to this study were purposively selected and physicians were randomly recruited to participate in focus group discussions. Nvivo version 10 was used to inductively code the qualitative transcripts and manage thematic data analysis.

**Results:**

Inappropriate antibiotic prescribing was a common practice and driven by seven factors: prescribing habit, limited diagnostic capacity, lack of microbiology evidence, non-evidence-based clinical guidelines, perceived patient demand, poor hygiene and infection control, and perceived bacterial resistance to narrow spectrum antibiotics.

*“Every day, doctors are not performing appropriately. We have made lots of mistakes with our antibiotic prescribing.”*

When a patient’s clinical condition was not responsive to empiric treatment, physicians changed to a broader spectrum antibiotic and microbiology services were sought only after failure of a treatment with a broad-spectrum antibiotic. This habitual empirical prescribing was a common practice regardless of microbiology service accessibility. Poor hygiene and infection control practices were commonly described as reasons for ‘preventive’ prescribing with full course of antibiotics while perception of bacterial resistance to narrow-spectrum antibiotics due to unrestricted access in the community resulted in unnecessary prescribing of broad spectrum antibiotics in private practices.

**Conclusions:**

The practice of prescribing antibiotics by Cambodian physicians is inappropriate and based on prescribing habit rather than microbiology evidence. Improvement in prescribing practice is unlikely to occur unless an education program for physicians focuses on the diagnostic capacity and usefulness of microbiology services. In parallel, hygiene and infection control in hospital must be improved, evidence-based antibiotic prescribing guidelines must be developed, and access to antibiotics in community must be restricted.

## Background

Soon after the introduction of penicillin for clinical treatment in 1940s Alexander Flaming expressed concern that physicians frequently failed to respect prescribing rules and warned of bacterial resistance to penicillin [1]. Nearly three decades ago the Infectious Diseases Society of America developed guidelines in an effort to improve antibiotic prescribing [2] and recently the World Health Organization (WHO) released a disturbing report of global inappropriate antibiotic use that is now in epidemic proportions [3]. In accordance with WHO the definition of inappropriate antibiotic use includes seven errors: over prescription, omission of prescription, incorrect selection, unnecessary expense, inappropriate dosage, incorrect route and incorrect duration [4]. Inappropriate antibiotic use is especially high in resource-poor countries and occurs in both healthcare and non-healthcare settings with physicians, patients and the general public accelerating the trend [5–7]. In resource-poor settings poor prescribing is driven by a complex combination of socio-behavioural and economic factors and a weak functioning healthcare system that is absent of the ability to enforce guidelines [8–10].

Like other resource-poor settings inappropriate antibiotic use [11, 12] and antibiotic resistance [13–15] in Cambodia are common place. Effective interventions require background knowledge of the phenomena that drive inappropriate antibiotic prescribing. We recently reported that over half of Cambodian physicians working in public hospitals surveyed nationally acknowledged that their antibiotic prescribing was inappropriate [16]. Following from this prescribing practice survey we used qualitative interviews to explore their antibiotic prescribing practices that may drive antibiotic resistance in Cambodia.

## Methods

### Study design and setting

This qualitative study used focus group discussions (FGDs) to collect data. Cambodia is a low-income country located in Southeast Asia with over 11 out of 15 million people being poor or near poor [17]. It was reported in 2011 that the Cambodian healthcare system employed 19,721 healthcare staff including 3,196 physicians working in 91 hospitals across the country [18].

### Sampling and data collection

Purposive sampling [19] was used to select hospitals that participated in a knowledge, attitude and practice (KAP) survey [16] of antibiotic prescribing prior to this current study and physicians were randomly selected from these facilities to participate in FGDs. Data collection occurred between September 2013 and February 2014. Participating physicians were invited to a meeting room in their hospital where they were given an information sheet and were consented to participate in FGDs. There were between four to 10 physicians in each FGD depending on the size of participating hospitals. A standardized prompting question guide and probing techniques were used in each FGD. Enrolment continued until data saturation was achieved when no new conceptual ideas or themes emerged to warrant further investigation [20]. All FGDs were digitally recorded.

### Data analysis

Each digital record of FGDs was transcribed verbatim into Khmer. A local physician was employed to check the accuracy of the transcripts against the audio to ensure correct transcribing of the medical terminology used by participating physicians. All edited Khmer texts were then translated into English and checked by CO. An inductive approach was used to code patterns or ideas that emerged from the data. Coding was conducted by two coders (CO and MM) and any unclear text were checked (CO) and any discrepancies between the two codes were discussed. Nvivo version 10 was used for coding and managing data analysis. Data were analysed using thematic data analysis techniques and presented as thematic syntheses and an illustrative visual display [21, 22].

## Results

We conducted 17 FGDs with 103 participating physicians from 11 public hospitals including four hospitals that had microbiology services. Our findings revealed that antibiotics prescribing occurred in the absence of microbiology evidence of infection.

*“Every day, doctors are not performing appropriately. We have made lots of mistakes with our antibiotic prescribing.” Ph#4-KCH*

*“Nowadays we prescribe antibiotics blindly. We just think that this is perhaps Gram negative or Gram positive. So we just give antibiotics to cover Gram positive or negative. But to make sure which one is correct or how many days we should give this or that type to patients, we don’t know.” Ph#2-MBR*

This empirical prescribing of antibiotics was common regardless of accessibility to microbiology services and facilitated by seven factors as reported below:

### Empirical prescribing habit

**Fig. 1:**
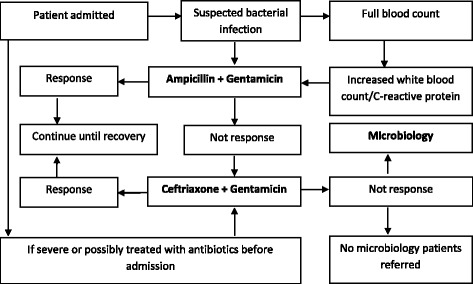
Habitual Pattern of Empirical Antibiotic Prescribing

When a suspected case of bacterial infections was not responsive to the initial antibiotic treatment the common and habitual approach was to change to a broader spectrum antibiotic without microbiology evidence. Amoxicillin, ampicillin, gentamicin, ciprofloxacin, and cotrimoxazole were routinely the initial choice but when the clinical condition was unresponsive physicians would change to a third generation cephalosporin (usually ceftriaxone) as this was the currently broadest spectrum beta-lactam antibiotic supplied in public hospitals. If the disease remained unresponsive to ceftriaxone, physicians working in hospitals without microbiology services would need to send patients to a referral hospital for further treatment (Fig. 1). When physicians perceived patients were severely ill or suspected the patients had received treatment prior to admission they were less likely to prescribe a narrow spectrum antibiotic.

*“When we suspect typhoid fever, we initially prescribe quinolone for 3 days. If the patients’ body temperature cools down, then we will continue for 5 days or 1 week more and then we stop. If the body’s temperature does not cool down after 3 days we will do another blood cell count. If we see something strange or the white blood cell count is getting high, then we will change to use ceftriaxone. If ceftriaxone is still not effective, then we will refer the patients to have culture.” Ph#3-KTL*

*“For me in the medical ward, I often give ceftriaxone because when I take the clinical history of patients, it always shows that they have already received treatment a few days from private clinic but they didn’t get better. They took antibiotics or we found antibiotics with them.” Ph#7-KSFH*

### Limited diagnostic capacity

Diagnostic uncertainty facilitated antibiotic prescribing. The most challenging was the difficulties in differentiating bacterial from viral infections and when physicians were uncertain about diagnosis their first option was an antibiotic rather than waiting several days to see if the patient’s condition improved.

*“When our diagnosis is not certain we can’t avoid antibiotics. For example, now we have a patient with sore throat, we are not sure if it is caused by virus or bacteria. We don’t know. The problem of uncertainty forces physicians to give antibiotics because if we don’t, we are afraid.” Ph# 4-KCH*

*“For me I think whether the prescription is correct or not, it relates to the capacity of physicians. For some physicians their prescriptions look inappropriate. There are no signs of an infection but still they prescribe antibiotics, and three types of antibiotics are prescribed. It relates to the capacity and knowledge of the physicians. When physicians think that they are incompetent, they prescribe antibiotics.” Ph#1-SRP*

Physicians focused on the ability to make the correct diagnosis in the absence of microbiology rather than the impact of an incorrect choice of antibiotics.

*“First make a correct diagnosis and then if the medicines are not working, that diagnosis is incorrect causing ineffective antibiotic therapy.” Ph#4-SRP*

### Absence of microbiology evidence of bacterial infection

Access to microbiology services remained scarce in Cambodia, especially for physicians working in provincial and district referral hospitals where empirical antibiotic prescribing was based solely on clinical presentation and prescribing experience.

*“We don’t have culture that is why we prescribe a trial of antibiotics for 3 days and if that is ineffective, we would change to other antibiotics.” Ph#2-KTL*

*“Firstly in severe cases we can’t wait and we have to fight the infection immediately (with antibiotics). We can’t wait (just) because we don’t have microbiology available to us, we can’t delay (treatment).” Ph#8-KP*

Empirical antibiotic prescribing was common even when microbiology services were available as diagnostic and antibiotic susceptibility testing were only sought when the broadest spectrum antibiotics, such as ceftriaxone, had failed to improve the patient’s condition (Fig. 1).

*“Here we have it [microbiology]…they [physicians] are not likely to request culture until the patient under treatment has not improved then they order it [culture]. For first line antibiotics, they rarely do it [culture]. Most of them practice like this. I think this is a habit from the previous time when there was no culture. Now, we have culture service but their habit does not change. It deeply rooted.” Ph#4-KCH*

Physicians’ reasons for not using microbiology services included prescribing habit, a perception that the time to results was excessive, culture results were unreliable or poor meaning results and clinical progression were discordant, and patient’s financial constraints.

*“Of course, I do not know why the lab takes so long; maybe it is about quality of lab that is not reliable. Because of that physicians don’t wait. As the baby now has very high temperature and in a bad condition, physicians are concerned about sepsis and afraid that the baby may die, so they stop giving ampicillin and gentamicin, they only prescribe ceftriaxone and add ciprofloxacin.” Ph#4-NPH*

*“For the culture, what is important is the patients and money. They don’t have money to continue their stay in hospital to wait for culture results that take long time. That’s why we often give the medicines right away. Generally speaking, the important thing is people don’t have money to follow our treatment rules.” Ph#6-KSM*

### Perceived non evidence-based clinical guidelines

At the time of the interviews all available clinical practice guidelines were not current.

*“We have [treatment guidelines] but most are old…there are no newly updated guidelines.” Ph#3-KCH*

*“No, we don’t have [treatment guidelines] yet in my [Infectious Diseases] ward…I want them so that the whole hospital can practice consistently.” Ph#5-NPH*

Some treatment guidelines were up-to-date but only for some specific diseases such as neonatal sepsis or normal vaginal delivery. Yet, antibiotic prescribing was based on habit rather than these guidelines. For instant, antibiotics were not indicated for normal vaginal delivery but every patient received a 5-day course of antibiotics and the treatment of neonatal sepsis was based on the individual physicians’ experience not the guidelines.

*“For women with a cesarean they receive (5 days of) injections of ampicillin, gentamicin or ceftriaxone. Depending on physicians, sometimes they give ceftriaxone, sometimes ampicillin, gentamicin. For normal delivery, we also use for 5 days.” Ph#6-SFH*

*“Well, it is heterogeneous…it varies from physician to physician. One physician does it differently from another without following the epidemiology, the disease in that locality, country service, or disease. They don’t have that basis.” Ph#4-NPH*

Physicians believed these treatment guidelines were based on those from developed countries where the standard of hygiene and infection control was high. Physicians wanted locally developed guidelines as they believed these would help them improve their prescribing.

*“In public hospitals, we want to change our attitude to just give prophylactic antibiotics. So we need a study.…....comparisons between prophylaxis and treatment and then show results to physicians so that they can try to prescribe only prophylaxis with narrow spectrum antibiotics to reduce resistance and the antibiotic budget; and it’s also easier because the staff don’t need to give injection every day.” Ph#6-KCH*

### Patient’s demand

Physicians described that patients commonly expected injections not tablets because they believed that injections were more effective. In private clinics patients asked physicians for ‘strong’ and ‘quality’ medicine because they wanted a quick cure to ensure they had a short clinic admission. Although patients in reality did not demand antibiotics physicians prescribed them to meet this perceived demand.

*“In private services, we think about what to do to make patients stay short and cure. This is the important point. If we spend the first 5 days on amoxicillin and there’s no improvement and then we change to cefexime for another 5 days. That’s too long for them and they still may not be cured.” Ph#2-SRP*

*“A wealthy family comes to us and asks for a ‘strong’ medication for their kid, and we would give them something like augmentin (amoxicillin and clavulanate potassium) from France and so on.” Ph#5NL*

*“Patients want ‘quality’ medicine. They don’t say ‘ceftri [ceftriaxone]’ they just say that they want ‘quality’ medicine. With stronger medicine, they believe they recover faster.” Ph#4-NPH*

Post-partum patients on discharge commonly asked for antibiotics in a belief that the antibiotics would heal their internal wounds and reduced their pain. Physicians fulfilled this request by prescribing an additional 5 days of antibiotics. This occurred in both a national speciality hospital and general hospitals.

*“Patients say they want a prescription to buy additional medicines to take to reduce pain or something like that. So we just prescribe one antibiotic, one analgesic, and one vitamin for all kinds of patients, like those who have normal delivery and so on.” Ph#2-NMCH*

### Perceived poor hygiene and infection control

**Fig. 2:**
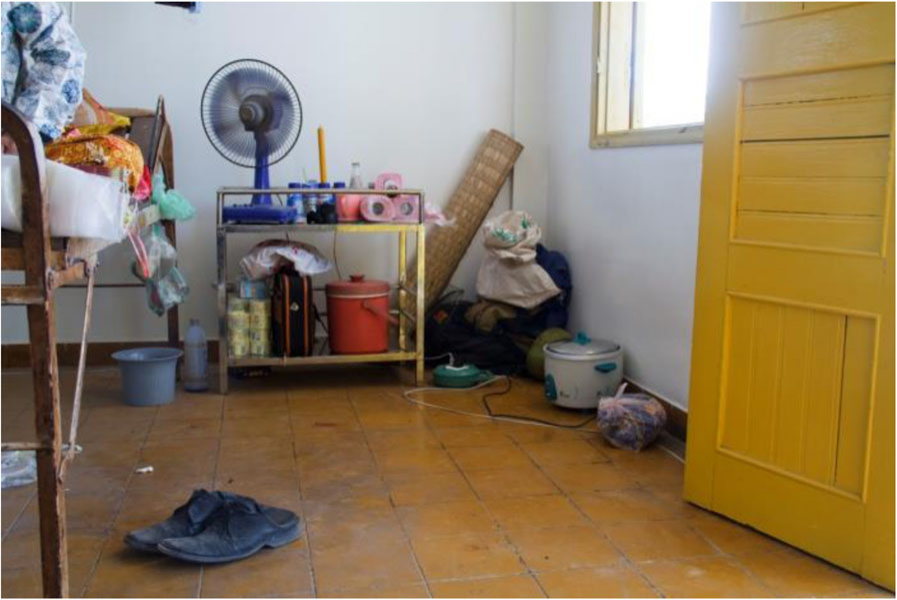
Patient room in medical ward of a provincial hospital

Patients’ rooms were commonly over-crowded, unclean and untidy (Fig. 2). Families provided the patients with bedding material, cooking utensils and food and assisted with the patient’s general hygiene. In addition, physicians perceived infection control practices were substandard, and community sanitation and patient personal hygiene were poor. Because of these challenges and their constant concern about infection transmission physicians prescribed antibiotics for ‘preventive purpose’.

*“We often prescribe for prevention. When we prescribe, we don’t know whether disease is cured by antibiotics or there is no infection. We don’t know. I’m always afraid because everything is not clean and the patients are not hygienic so we give antibiotics right away.” Ph#4-KSM*

*“Yes. Normally even though we know that it is viral [infection], we still prescribe amoxicillin or something else. Why? We think that the environment outside is not good as there is a lots of flying dust.” Ph#1-KRV*

*“Patients’ clothes are not clean. So we use antibiotics because we are afraid of them getting an infection.” Ph#3-KP*

Physicians were concerned about wound cleaning, sterilization of surgical instruments and contaminated hands and prescribed antibiotics to ‘prevent’ complications.

*“We think our practice doesn’t comply with infection control guidelines. Before delivering a baby, we use gloves but we touch vaginal tears, touch everything. It is not appropriate. So we haven’t respected the infection control guidelines. When we know that our practices are not correct we use antibiotics immediately, and this continues over and over.” Ph#6-KCH*

*“We aren’t confident in the way staff clean wounds. And are we confident that sterilization is done correctly? Are the materials correctly sterilised? And the other thing is the responsibility of staff. We acknowledge they are less responsible. These are factors that worry us and we always think that we don’t want to make things more complicated. That’s why we have to use antibiotics to prevent infection so we can sleep well [no complication].” Ph#5-SRP*

### Perceived resistance to narrow spectrum antibiotics

Although physicians described their prescribing for ‘preventive’ purposes they also believed that pathogens were resistant to narrow spectrum antibiotics as a result of unrestricted antibiotic access in the community. Prior to consulting with a physician, patients self-administered a small dose of antibiotics purchased from a community pharmacy or drug outlet, or sought treatment from community-based healthcare providers.

*“Patients come through a healthcare provider at a community center, they also come through the untrained village healthcare provider who goes from house to house, and if they don’t get better they would turn to us [physician]. When they come to us, if we use ampicillin, it seems useless.” Ph#1-KP*

*“From day to day problem [antibiotic resistance] persists and with antibiotics prescribed in private practice we prescribe cefixime, 3* ^*rd*^ *generation cephalosporin, which is now considered to be a very low level antibiotics, as low as ampicillin.” Ph#9-KCH*

The perception of resistance to narrow spectrum antibiotics encouraged physicians to prescribe broad spectrum antibiotics so that a multitude of perceived resistant pathogens could be covered. Physicians then believed that they could keep their reputation and their private practice business. This prescribing in private practice included the latest group of fluoroquinolones and cephalosporins.

*“If it’s a broad spectrum, it can shoot better. Therefore, there are more chances of success.” Ph#2-NPH*

*“We also think about business because we run a business. If our treatment is not effective or it’s just similar to the treatments of the other [physicians] they [patients] don’t come to us again later.” Ph#1-KRV*

*“In my private place I always provide injections of ceftriaxone for patients before cesarean and five more doses later. For normal delivery I used simple pill. I used curam [amoxicillin and potassium clavulanate] for five days. Because I think that in Cambodia now ampicillin and something like that are all resistant.” Ph#5-NMCH*

## Discussion

In our previous national survey on the antibiotic prescribing practices using case presentations and attitudes over half of our physicians acknowledged that their prescribing was inappropriate [16]. In this current study we can confirm that antibiotic prescribing is inappropriate and one important factor is the practice of habitual empirical prescribing. There are several other driving factors, many of which are similar to other low resource settings [10, 23, 24]. Habitual prescribing has been observed elsewhere [24–28] and may be an important driver of global inappropriate antibiotic prescribing. To reduce unnecessary prescribing an intervention should start with changing habitual prescribing practices [26]. Our physicians’ inability to distinguish between bacterial and viral infections drives them to inappropriately prescribe an antibiotic which is also a global phenomenon [10, 23]. A study in India reported that such diagnostic uncertainty is a reason physicians prescribed antibiotics inappropriately [29]. A barrier to evidence-based prescribing in resource-poor countries is the lack of access to and inappropriate use of microbiology services [10]. A study in Bangladesh revealed that over 90% of antibiotic prescribing was empirical due to lack of access to and unreliable results of microbiology test [30]. But habitual prescribing can be so entrenched that even providing microbiology services may not change a physician’s prescribing if they choose not to order an antibiogram [27]. Physicians in India were reported to seldom request microbiology testing though accessible yet their prescribing was mainly empirical [31]. Underuse of or lack of access to microbiology services results in indiscriminate prescribing of broad spectrum antibiotics [32]. From our field observations, Cambodian physicians have very little appreciation about the usefulness of microbiology services and these are therefore under-utilized. They have little knowledge about what specimens to collect and the method of collection that will prevent the specimen from being contaminated. A Gram stain is a rapid and inexpensive test that can be utilized to assist prescribing while waiting for an antibiogram. Yet, our physicians do not know of this benefit which would reduce habitual and empiric prescribing. Training physicians about how to use microbiology may improve the use of microbiology services and improve antibiotic prescribing [33].

Poor hygiene and infection control practices encouraged our physicians to prescribe antibiotics excessively for preventive purpose although they were never certain about the outcomes of their prescribing in these circumstances. It is a common fear in resource-poor settings that poor infection control and environmental cleanliness will result in patients acquiring a healthcare associated infection and this encourages physicians to prescribe antibiotics [9, 10, 23]. Hygiene and infection control are not a priority in Cambodian hospitals and environmental cleaning and waste management are performed by illiterate staff who are not receive exceptional training [34].

Our previous survey reported over half of Cambodian physicians preferred to prescribe broad-spectrum antibiotics in their private practices [16]. Our physicians in this current study described how they preferred prescribing broad spectrum antibiotics in their private practice because they perceived that the unrestricted access to antibiotics in the community resulted in bacterial resistance to narrower spectrum antibiotics. Several studies have shown that in reality patients actual demand antibiotics or physicians just perceive this demand [10, 24, 29]. Our current study has described a similar perception with post-partum patients requesting antibiotics thinking that this will reduce pain and heal internal wounds. Physicians also described their private practice patients demanded ‘strong’ and ‘quality’ medicines resulting in broad spectrum antibiotics being prescribed to meet this perceived demand.

## Conclusions

The practice of prescribing antibiotics by Cambodian physicians is inappropriate and based on prescribing habit rather than microbiology evidence. Improving antibiotic prescribing is unlikely to occur unless education programs are provided to improve the diagnostic capacity and the usefulness of microbiology services. In the meantime antibiotic therapeutic guidelines should be developed from the limited microbiology services that are currently available. In parallel, hygiene and infection control in hospital must be improved, and access to antibiotics in community must be restricted.

## Abbreviations

FGD: Focus group discussion
UNSW: University of New South Wales
WHO: World Health Organization
